# Predictors of In-Hospital Mortality for School-Aged Children with Severe Traumatic Brain Injury

**DOI:** 10.3390/brainsci11020136

**Published:** 2021-01-21

**Authors:** Chih-Chi Chen, Carl P. C. Chen, Chien-Hung Chen, Yu-Wei Hsieh, Chia-Ying Chung, Chien-Hung Liao

**Affiliations:** 1 Department of Physical Medicine and Rehabilitation, Chang Gung Memorial Hospital, School of Medicine, Chang Gung University, 5 Fuhsing St., Taoyuan 333, Taiwan; Claudia5477@gmail.com (C.-C.C.); Carlchendr@gmail.com (C.P.C.C.); b9002052@cgmh.org.tw (C.-H.C.); chiaying928@yahoo.com (C.-Y.C.); 2 Department of Occupational Therapy and Graduate Institute of Behavioral Sciences, School of Medicine, Chang Gung University, 259, Sec1, WenHua First Road, Taoyuan 333, Taiwan; Ywhsieh@mail.cgu.edu.tw; 3 Healthy Aging Research Center, Chang Gung University, 259, Sec1, WenHua First Road, Taoyuan 333, Taiwan; 4 Department of Trauma and Emergency Surgery, Chang Gung Memorial Hospital, School of Medicine, Chang Gung University, 5 Fuhsing St., Taoyuan 333, Taiwan

**Keywords:** school-aged children, traumatic brain injury, predictors, mortality

## Abstract

Traumatic brain injury (TBI) is the leading cause of mortality in children. There are few studies focused on school-aged children with TBI. We conducted this study to identify the early predictors of in-hospital mortality in school-aged children with severe TBI. In this 10 year observational cohort study, a total of 550 children aged 7–18 years with TBI were enrolled. Compared with mild/moderate TBI, children with severe TBI were older; more commonly had injury mechanisms of traffic accidents; and more neuroimage findings of subarachnoid hemorrhage (SAH), subdural hemorrhage (SDH), parenchymal hemorrhage, cerebral edema, and less epidural hemorrhage (EDH). The in-hospital mortality rate of children with severe TBI in our study was 23%. Multivariate analysis showed that falls, being struck by objects, motor component of Glasgow coma scale (mGCS), early coagulopathy, and SAH were independent predictors of in-hospital mortality. We concluded that school-aged children with severe TBI had a high mortality rate. Clinical characteristics including injury mechanisms of falls and being struck, a lower initial mGCS, early coagulopathy, and SAH are predictive of in-hospital mortality.

## 1. Introduction

Traumatic brain injury (TBI) is among the leading causes of mortality and morbidity in children and is an important global health concern [1,2,3,4]. Recently, U.S. Centers for Disease Prevention and Control reported that about 7% of 0–14 year old TBI children died after hospitalization for TBI [5]. In previous studies, mortality rate has been reported from 9% to 25% in severe pediatric TBI [6]. Early recognition of children with a high risk of mortality is important for clinicians to identify therapeutic options.

Pediatric TBI covers a wide range of age groups with different mechanisms of injury and mortality rate [7]. Abusive trauma is particularly common in children less than two years old [8]. Children younger than four years old had the highest fall-related head injury rate. Being struck by/against an object most commonly occurred in 5–14 year old children and traffic accidents most commonly above 15 years old [9,10]. Mortality rate was also different in various age groups. A recent study suggested that age less than 2 years was an independent predictor of mortality in severe head trauma patients [11].

Predictors to outcomes for pediatric TBI have been studied [12,13,14,15,16,17,18]. Most of these studies included all age groups of pediatric patients, and even especially focused on younger children (<4 years) [13,16]. Age-related structural changes, differences in the mechanisms of injury based on the child’s physical abilities, and the difficulty in performing neurological evaluations of younger pediatric populations contribute to the differences in the clinical characteristics of school-aged children and younger children with TBI [7]. Currently, studies focused on predictors of mortality in school-aged children are limited.

In this study, we analyzed the clinical characteristics of school-aged children with mild/moderate and severe TBI. We further derived predictors of in-hospital mortality in severe TBI. Our aim was to assist clinical doctors in identifying high-risk patients from the available information commonly collected in the emergency department (ED).

## 2. Materials and Methods

### 2.1. Study Design

This was a retrospective, observational cohort study conducted in a tertiary trauma center in Northern Taiwan. Children aged 7–18 years old who were admitted from January 2005 to December 2014 with TBI were enrolled. Patients were eligible if they were assigned the International Classification of Diseases, Ninth Edition (ICD-9) (WHO 1977) diagnostic codes 850–854 for intracranial injury. A physician and a research nurse reviewed all medical records. Patients who died at the scene or during transportation were excluded. Patients with prior central nervous system disorders were also excluded due to difficulty in assessing the outcomes of interest. Medical histories were gathered and reviewed during admission and noted in patient charts. Those with final diagnoses not related to cranial injury and those with an undetermined injury mechanism were excluded. Patients whose primary injury was extracranial despite having an ICD-9 code for intracranial injury were also excluded.

Patients were classified into groups with mild to moderate or severe TBI according to the severity of their injury. Injury severity was stratified by the initial Glasgow coma scale (GCS) score in admission. The GCS, which ranges from 3 to 15, was routinely scored by emergency medical service personnel at the emergency department. Severe TBI was defined by an initial GCS score ≤8. Mild to moderate TBI was defined by an initial GCS score >8. The Institutional Review Board at Chang Gung Memorial Hospital approved this study: IRB no. 20200050B0.

### 2.2. Variable Definitions

Data were extracted from the medical records of all eligible subjects. The outcome was defined as in-hospital mortality. The parameters selected for the evaluation of differences between pediatric patients with mild to moderate TBI and severe TBI included sex, age, mechanism of head injury and intracranial computed tomography (CT) findings in the ED, surgical intervention, and mortality. The parameters selected as potential predictors of mortality in patients with severe TBI included sex; age; mechanism of head injury; initial clinical presentation in ED, including the motor component of the GCS (mGCS); hypotension; hypothermia; initial laboratory data showing hyperglycemia; coagulopathy; and intracranial CT findings in the ED. We selected these parameters based on previous studies [11,19,20,21,22,23,24,25,26,27,28] that suggested that these were potential risk factors for injury severity and mortality in pediatric head injury patients.

The mechanisms of injury were recorded and classified into the following categories: falls, being struck by/against an object, traffic collisions, assault, and sports [29]. The mGCS was scored from 1 to 6. Previous studies have shown that the motor component alone is equivalent to full GCS with regard to the prediction of survival to hospital discharge [30]. Hypotension was defined as a systolic blood pressure (SBP) below the fifth percentile for age according to the Pediatric Advanced Life Support (PALS) guidelines from the American Heart Association for Cardiopulmonary Resuscitation and Emergency Cardiovascular Care. The formulas were as follows: <70 mmHg + (2 age in years) in children 1 to 10 years and <90 mmHg in children ≥10 years of age [31]. Hypothermia was defined as an initial body temperature below 35 °C [32], early coagulopathy was defined as an international normalized ratio (INR) ≥1.2 [19,32], and hyperglycemia was defined as a blood glucose level greater than 200 mg/dL [11] on admission to the ED.

Pediatric radiologists reviewed the initial noncontrast CT scans obtained in the ED at admission. Specific types of intracranial injuries, including cranial bone fracture, subdural hematoma (SDH), epidural hematomas (EDH), parenchymal hemorrhage, subarachnoid hemorrhage (SAH), cerebral edema, and mass effect, were identified and reported [33,34].

### 2.3. Statistical Analysis

Descriptive statistics were used to identify differences in clinical characteristics and outcome variables among patients with mild to moderate and severe head trauma. For categorical variables, comparisons were made using Pearson’s chi-square tests or Fisher exact test if any expected cell size was less than 5. The Mann–Whitney U test was used to compare the continuous variable distribution including age and mGCS. The missing observations were estimated using multiple imputation assuming they were missing completely at random. This technique permits the analysis of complete data sets in which missing values are filled in based on regression-predicted values by generating multiple complete data sets [35]. To identify potential predictors of in-hospital mortality among school-aged children with severe TBI, multivariate logistic regression analyses were conducted. The candidate predictors with a significant level of *p* < 0.1 were identified by univariate analysis. A *p*-value < 0.05 was considered statistically significant for the multivariate analyses. We used the receiver operating characteristic (ROC) to evaluate the ability to predict poor outcomes by the identified potential predictors in multivariate analysis. All statistics were performed using STATA version 14.0 software (STATA, Inc., College Station, TX, USA).

## 3. Results

**Figure 1:**
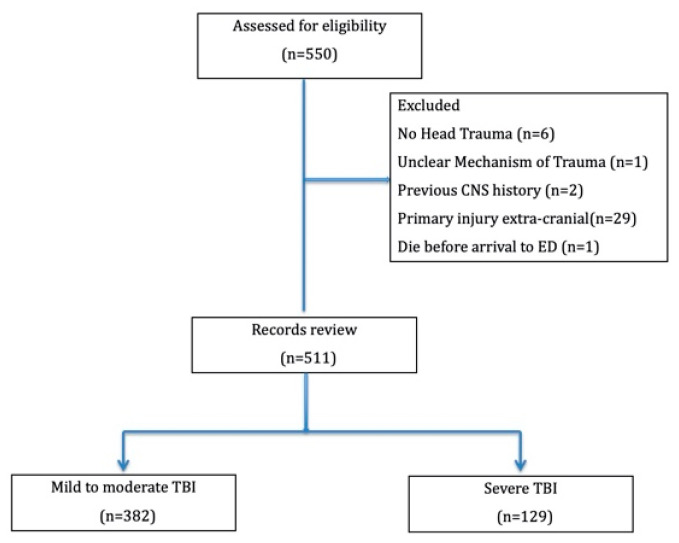
Inclusion and exclusion flow chart.

Of the 550 initially identified children aged between 7 and 18 years, six were excluded because they had a final diagnosis other than TBI, two were excluded due to a previous central nervous system disease (one with cerebral palsy, one with a history of a brain operation), 29 were excluded because they had a primary extracranial injury, and one was excluded because the mechanism of injury was unknown. The resulting study population consisted of 511 patients, including 382 (74.6%) with mild to moderate TBI and 129 (25.2%) with severe TBI (Figure 1).

A total of 511 school-aged children with TBI were enrolled in this study. (Table 1) Most of the children were boys (70.1%). Their median age was 16 years old. Traffic accidents were the most common mechanism of injury (70.8%), followed by falls (19.6%), being struck by/against an object (6.9%), assault (1.6%), and sports (1.2%). A total of 146 (28.6%) children received a neurosurgical intervention. Thirty-two children (6.3%) died in the hospital. Hyperglycemia had the most missing values (26.4%), followed by coagulopathy (7.8%), hypotension (1.11%), and hypothermia (1.01%).

Compared to children with mild to moderate TBI (Table 1), children with severe TBI were older (*p* < 0.001) and more commonly had injury mechanisms of traffic accidents (*p* < 0.001). Their intracranial radiology findings showed more SDH (*p* < 0.001), SAH (*p* < 0.001), parenchymal hemorrhage (*p* < 0.001), cerebral edema (*p* < 0.001), and mass effect (*p* < 0.001), and less EDH (*p* = 0.001). Patients with severe TBI were more likely to need surgical intervention (*p* < 0.001), and their mortality rate was higher (*p*< 0.001) than those with mild to moderate TBI. There was no significant difference in sex, age distribution, and CT evidence of skull fracture between children with mild to moderate and severe TBI.

Among the children who presented with severe TBI (*n* = 129), thirty children (23.3%) died in the hospital. (Table 2)

The mechanism of injury was significantly associated with mortality (*p* = 0.024). Among patients with severe TBI, being struck by/against an object and assault were associated with higher mortality rates (3 of 4 and 2 of 3, respectively). Other clinical variables, including hypothermia (*p* = 0.007), hypotension (*p* = 0.047), lower mGCS score (*p* < 0.001), early coagulopathy (*p* < 0.001), and intracranial radiology findings of SAH (*p* = 0.012), were all associated with higher mortality rates. No significant differences in gender, age distribution, initial glucose level, or CT evidence of skull fracture, SDH, EDH, parenchymal hemorrhage, cerebral edema, or mass effect were detected between nonsurviving and surviving children with severe TBI.

In multivariate analyses (Table 3) of the potential predictors of in-hospital mortality among school-aged children with severe TBI, the mechanism of injury, hypothermia, hypotension, mGCS score, early coagulopathy, initial hyperglycemia, SAH, and cerebral edema were evaluated. Falls and being struck by/against an object were associated with significantly increased odds of mortality in severe TBI patients when compared to traffic accidents (adjusted OR = 19.66, 95% CI: 1.16–334.25, *p* = 0.039 and adjusted OR = 98.97, 95% CI: 3.09–3167.04, *p* = 0.009, respectively). Children with lower initial mGCS scores had a higher mortality rate. Each one-point decrease in the mGCS score was associated with a 3.22-fold increase in the odds of mortality (adjusted OR = 3.17, 95% CI: 1.9–5.29, *p* < 0.001). An initially elevated prothrombin time (OR 5.68, 95% CI = 1.08–29.81, *p* = 0.040) and CT evidence of SAH (OR 5.22, 95% CI = 1.28–21.26, *p* = 0.021) also independently predicted mortality.

The area under the receiver operating characteristic curve for the multivariate model, including the mechanism of injury, mGCS score, early coagulopathy, and CT finding of SAH, was 0.905.

## 4. Discussion

This study identifies the predictors of mortality in school-aged children with severe TBI, which are focused on less in current literature [11,36]. Our results showed that approximately 25% of school-aged children with TBI arrived at the ED with severe TBI. In comparison to children with mild to moderate TBI, children with severe TBI were older. Their mechanism of injury was more commonly related to traffic accidents. Patients with severe TBI had more SDH, SAH, parenchymal hemorrhage, cerebral edema, and mass effect, and less EDH. They were more in need of neurosurgical intervention and had a higher in-hospital mortality rate. The mortality rate of school-aged children with severe TBI was 23% in this study, which was similar to that reported in a recent study in the US (22.8%) [37]. Our study further showed that injury mechanisms of falls, being struck by/against an object, having a lower mGCS score, an initial elevated prothrombin time, and CT findings of SAH were independent predictors of mortality.

As in a previous study, our study showed that boys are more likely to experience TBI than girls among school-aged children [4]. However, sex was not associated with differences in TBI severity or mortality. More than half of the school-aged children admitted for TBI were older than 16 years old. A bimodal age distribution in TBI has often been described in previous studies. Young children (0–2 years) and adolescents (15–18) are more commonly injured [4]. Furthermore, those admitted with severe TBI tended to be older than those admitted with mild to moderate TBI. Such findings may be related to a higher frequency of traffic accidents among older groups. However, although older age was associated with increased severity of TBI, it was not associated with increased mortality. A recent study by Sarnaik et al. also suggested that there were no differences in mortality among age groups in children with severe TBI [38].

As previously mentioned, traffic accidents were the most common mechanism of injury in these school-aged children in our study. Dewan et al. also suggested that motor vehicle collisions were responsible for a larger proportion of pediatric TBIs in high-income countries [4]. Specific mechanisms of TBI were also associated with the severity of the TBI. In our study, children with TBI caused by traffic accidents were more likely to have severe TBI. Traffic accidents were the leading cause of mortality. A recent study in the U.S. also found that motor vehicle crashes were the leading cause of death in children and adolescents [3,38]. Traffic accident prevention measures are warranted in school-aged children. The incidence of injury mechanism of being struck by/against an object, assault, and sports were low in our study. Cultural differences may result in the broad range of mechanisms of pediatric TBI across population groups [4]. Even though the injury mechanisms of being struck by/against an object and assault were less common, they were related to higher mortality rates than traffic accidents among children with severe TBI and were independent predictors of mortality. Although the cause is unclear, it is probably multifactorial and includes factors such as different mechanisms of head injury and delays in seeking medical assistance, leading to deterioration of the patient’s condition.

The causes of hypothermia are multifactorial and not only related to the traumatic event directly but also to the body’s systemic response to the injury [39]. Jennifer et al. suggested that hypothermia is associated with increased mortality in pediatric trauma patients [40]. A recent study by Tude Melo et al. further suggested that accident-induced hypothermia is an independent predictor of mortality in children less than 17 years old with severe TBI. In our study, children with severe TBI who initially presented with hypothermia had an elevated mortality rate, but hypothermia was not an independent predictor of mortality in the multivariate analysis. These differences may relate to different age groups in our study and in that performed by Tude Melo et al. While our study focused on children above 6 years old and found that only 5% of children with severe TBI had hypothermia, Tude’s study recruited children from birth to 17 years old and showed that 17% of the children had hypothermia. A previous study on the epidemiology of accident-induced hypothermia in children suggested that hypothermia is less common in children older than 6 years old [41].

Hypotension was associated with increased mortality in our study but was not an independent predictor of mortality. A retrospective study in general pediatric TBI patients (<16 years old) suggested that hypotension was an independent predictor of mortality with a relatively low odds ratio (OR: 1.5) compared to those without hypotension [19]. Adequate SBP is important for maintaining cerebral blood perfusion in TBI patients, the regulation of which was even more impaired in younger children whose baseline blood pressure was even lower than that of older children [42]. Our study focused on older children, which may explain the lower influence of hypotension on mortality.

Concerns about the difficulty of assessing the full GCS have been reported due to the possibility of poor verbal communication skills in pediatric patients, aphasia, or ventilator use [43]. The mGCS score has been suggested as a tool for the evaluation of TBI instead of the full GCS. Van de Voorde P. el at. collected data from 96 children with moderate to severe TBI (0–18 years) and suggested that the mGCS had the same predictive ability for disability and mortality as the total GCS [21]. Shannon et al. reviewed 2341 TBI children younger than 18 years and stated that the mGCS score is equivalent to the full GCS score with regard to the prediction of the need for craniotomy, survival to hospital discharge, or dependence on a caretaker at follow-up [22]. One recent systematic review and meta-analysis article compared the total GCS with the mGCS and suggested that differences in the prediction of in-hospital mortality based on the total GCS score and the mGCS score were small and likely to be clinically unimportant. The mGCS is convenient and easy to use [44]. Our study also confirmed that the mGCS score is an independent predictor of mortality in school-aged children with severe TBI. For every one-point decrease in the mGCS score, the odds of mortality increased 3-fold.

Hyperglycemia commonly occurs in pediatric patients with moderate to severe TBI, and the incidence has been reported to be as high as 44% [23]. A previous study suggested that hyperglycemia is a transient phenomenon and cannot be used to predict mortality in pediatric TBI patients [45]. In contrast, an association with increased mortality in severe TBI patients has been reported in other studies [23,24,46]. In our study, there was no significant association between initial hyperglycemia and mortality in children with severe TBI. Rebecca L. Smith suggested that in children with severe TBI, hyperglycemia lasting longer than 48 h is associated with a poor outcome [23]. Our study focused on initial hyperglycemia and did not find an association. This result was compatible with that of another study that evaluated the relationship between hyperglycemia and mortality in children with TBI aged <16 years with GCS scores ≤13, which suggested that although there was a strong association between initial hyperglycemia and mortality in these patients, the same association was not observed in patients with GCS scores ≤8 [24]. In addition to the different timing of the assessment of hyperglycemia and injury severity, hyperglycemia was reported to be more common in children with TBI who were younger than 4 years [47]; this age group was not included in our study. A recent meta-analysis suggested that achieving glycemic control does not improve mortality but increases the incidence of hypoglycemia in patients with TBI [48].

Trauma-induced coagulation (TIC) has been commonly observed in trauma patients. The classic mechanism underlying TIC includes hypothermia, acidosis, hemodilution, and consumption of coagulation factors secondary to local activation of the coagulation system following severe traumatic injury [18]. TIC seems to be less common in pediatric than adult trauma patients. In children, TBI may also induce coagulopathy. Coagulopathy has been found to be associated with increased mortality in pediatric TBI patients [19,20,25]. Our study also suggested that initial coagulopathy was an independent predictor of mortality in these school-aged patients with severe TBI. Early monitoring of a patient’s coagulation profile can be used to predict the outcome. The mechanism underlying the development of TIC after TBI in pediatric patients has not been well studied, and currently, no study has shown that the correction of TIC can improve the outcome in these children [19].

EDH was more common in children with mild and moderate TBI than in those with severe TBI in our study. Such findings are compatible with those of Gerlach’s study, which followed 39 consecutive children and adolescents and found that EDH was associated with a good outcome [49]. The better outcome was related to the rarity of EDH being associated with intracranial injury and the early diagnosis and initiation of appropriate treatment [50]. Despite its relatively better outcome, EDH is still potentially life-threatening, with a reported mortality rate of up to 4.8% in young children [26]. SDH, SAH, Parenchymal hemorrhage, cerebral edema, and mass effect were all associated with increased severity of TBI. Among these abnormal neuroimaging findings, SAH was associated with increased mortality in children with severe TBI. Pillai S et al. analyzed 74 children aged less than 15 years old with severe diffuse brain injury and found that SAH was associated with a poor discharge outcome (mortality and vegetative status) [27]. In 2014, Hochstadter et al. analyzed 171 severe TBI patients aged less than 18 years with and without multisystem trauma and suggested that the presence of SAH was indicative of more severe TBI and a higher mortality rate but was not an independent predictor of mortality [28]. In our study, SAH was an independent predictor of mortality. A previous large cohort study in adult patients with severe TBI also suggested that SAH was an independent predictor of mortality [33]. The differences between our study and that performed by Hochstadter et al. may be related to the inclusion of different patient populations. We only included children aged 7–18 years and excluded those with major extracranial injury. Predictors of mortality that may be related to extracranial injury, such as liver laceration-induced blood loss and other associated physiologic changes, may not have been reflected in our study [51,52].

### Limitation

This study has some limitations. First, our results were based on a hospital-based registry, limiting the generalizability of the sample population. A study with a larger sample size would be more representative of the population. Second, rapid change of TBI severity from initial evaluation may occur for the clinically well-known dynamic evolution in these patients. The time lag (usually less than 30 min in our hospital) between the initial evaluation and neuroimage assessment may also be a confounding factor in the accurate classification of TBI severity. Third, some patient data, including blood glucose levels and prothrombin time, were missing. We used multiple imputation instead of complete data analysis to reduce the selection bias based on recent studies that suggested that valid multiple imputation reduced bias even when the proportion of missing data was large [53]. Fourth, not all potential predictors for in-hospital mortality were examined in this study, as we only assessed routinely documented and well-recorded clinical characteristics. Detailed recordings about the magnitude of the accidents and vehicles involved, use of helmet or not, intoxication status (alcohol and drug), and electrolyte imbalance [54] were lacking. Further prospective cohort studies are warranted to clarify the clinical presentation, outcomes, and prognostic factors in school-aged children with TBI.

## 5. Conclusions

School-aged children with different severities of TBI had varied clinical presentations and mortality rates. We identified the initial clinical characteristics that are predictive of a higher mortality rate in school-aged children with severe TBI. These predictors include injury mechanisms of fall and being struck by/against an object, initial low mGCS score, early coagulopathy, and CT evidence of SAH. Clinicians should be alert to the higher risk of mortality in these children on admission.

## Figures and Tables

**Table 1:** Demographic and clinical characteristics of mild–moderate and severe traumatic brain injury.

Clinical Characteristics	Total (*n* = 511)	Mild–Moderate TBI (*n* = 382)	Severe TBI (*n* = 129)	*p* Value
**Patient Characteristics:**				
**Gender, *n* (%)**				
Boys	358 (70.1)	266 (69.6)	92 (71.3)	0.718
Girls	153 (30.0)	116 (30.4)	37 (28.7)	
**Age (years)**				
Median (25, 75%)	16 (12,18)	16 (11,17)	17 (16,18)	<0.001
**Mechanism of Injury, *n* (%)**				
Fall	100 (19.6)	93(24.4)	7(5.4)	0.000
Struck by/against an object	35 (6.9)	31(8.1)	4(3.1)	
Traffic accident	362 (70.8)	247(64.7)	115(89.2)	
Assault	8 (1.6)	5(1.3)	3 (2.3)	
Sport	6 (1.2)	6(1.6)	0(0)	
**Cranial bone fracture, *n* (%)**				
Present	35 (6.9)	24 (6.3)	11 (8.5)	0.383
Not present	476 (93.2)	358 (93.7)	118 (91.5)	
**Subdural hemorrhage, *n* (%)**				
Present	141 (27.6)	87 (22.8)	54 (41.9)	<0.001
Not present	370 (72.4)	295 (77.2)	75 (58.1)	
**Epidural hemorrhage, *n* (%)**				
Present	120 (23.5)	104 (27.2)	16 (12.4)	0.001
Not present	391 (76.5)	278(72.8)	113 (87.6)	
**Parenchymal hemorrhage, *n* (%)**				
Present	106 (20.7)	56 (14.7)	50 (38.8)	<0.001
Not present	405 (79.3)	326 (85.3)	79 (61.2)	
**Subarachnoid hemorrhage, *n* (%)**				
Present	149 (29.1)	56(43.4)	93 (24.4)	<0.001
Not present	362 (70.8)	73 (59.6)	289 (75.7)	
**Cerebral edema, *n* (%)**				
Present	48 (9.4)	14 (3.7)	34 (26.4)	<0.001
Not present	463 (90.6)	368 (96.3)	95 (73.6)	
**Mass Effect, *n* (%)**				
Present	48 (9.4)	21 (5.5)	27 (20.9)	<0.001
Not present	463 (90.6)	361 (94.5)	102(79.1)	
**Neurosurgical intervention, *n* (%)**				
Yes	146 (28.6)	70 (18.3)	76 (58.1)	<0.001
No	365 (71.4)	312 (81.7)	53 (41.0)	
**Mortality, *n* (%)**				
Yes	32 (6.3)	2(0.52)	30 (23.3)	<0.001
No	479 (93.7)	380 (99.5)	99(76.7)	

TBI: Traumatic brain injury.

**Table 2:** Univariate analyses of association of mortality in school-aged children with severe traumatic brain injury.

	Alive, *n* (%)	Died, *n* (%)	Test Statistic	*p* value
**Patient Characteristics:**				
**Gender**				
Boys	72 (78.3)	20 (21.7)	0.41	0.520
Girls	27 (73.0)	10 (27.0)		
**Age (years)**				
Median (25, 75%)	17 (16,18)	17 (16,18)		0.581
**Mechanism of Injury**				
Fall	6 (85.7)	1 (14.3)	9.85	0.024
Struck by/against injury	1 (25.0)	3 (75.0)		
Traffic accident	91 (79.1)	24 (20.9)		
Assault	1 (33.3)	2 (66.7)		
**Clinical Presentations:**				
**Hypothermia**				
Present	2 (28.6)	5 (71.4)	10.05	0.007
Not present	97 (80.2)	24 (19.8)		
**Hypotension**				
Present	3 (42.9)	4 (57.1)	4.99	0.047
Not present	92 (79.3)	24 (29.7)		
**M component of GCS**				
M5 (Localizes to pain)	49 (90.7)	5 (9.3)	60.19	<0.001
M4 (Withdrawal to pain)	34 (91.9)	3 (8.1)		
M3 (Abnormal flexion to pain)	9 (100)	0 (0)		
M2 (Extension to pain)	3 (42.9)	4 (57.1)		
M1 (No motor response)	4 (18.2)	18 (81.8)		
**Prothrombin time**				
>1.2	43 (63.2)	25 (36.8)	15.4	<0.001
≤1.2	48 (94.1)	3 (5.9)		
**Glucose**				
>200	14 (63.60)	8 (36.4)	3.38	0.066
≤200	60 (82.2)	13 (17.8)		
**Cranial bone fracture**				
Present	9 (92.0)	2 (8.0)	0.17	0.506
Not present	90 (94.9)	28 (5.1)		
**Subdural hemorrhage**				
Present	38 (70.4)	16 (29.6)	2.11	0.146
Not present	61 (81.3)	14 (18.7)		
**Epidural hemorrhage**				
Present	13 (81.2)	3 (18.8)	0.21	0.762
Not present	86 (76.1)	27 (23.9)		
**Parenchymal hemorrhage**				
Present	38 (76)	12 (24)	0.025	0.874
Not present	61 (77.2)	18 (22.8)		
**Subarachnoid hemorrhage**				
Present	37 (66.1)	19 (33.9)	6.32	0.012
Not present	62 (84.9)	11 (15.1)		
**Cerebral edema**				
Present	22 (64.7)	12 (35.3)	3.75	0.053
Not present	77 (81)	18 (19)		
**Mass Effect**				
Present	18 (66.7)	9 (33.3)	1.94	0.163
Not present	81 (79.4)	21 (20.6)		

GCS: Glasgow Coma Scale.

**Table 3:** Multivariate predictive models for severe traumatic brain injury in school-aged children.

	Adjusted OR	95% CI	Z Score	*p* Value
Mechanism of injury				
Fall	19.66	1.16–334.25	2.06	0.039
Struck by/against an object	98.97	3.09–3167.04	2.60	0.009
Traffic accident	1			
Assault	3.045	0.01–1369.84	0.36	0.721
Hypothermia	2.45	0.10–60.92	0.55	0.586
Hypotension	1.74	0.24–12.60	0.55	0.586
Motor component of GCS	3.17	1.90–5.29	4.42	<0.001
Prothrombin time	5.68	1.08–29.81	2.05	0.040
Hyperglycemia	1.08	0.16–7.19	0.08	0.936
Subarachnoid hemorrhage	5.22	1.28–21.26	2.31	0.021
Cerebral edema	0.43	0.09–2.07	−1.05	0.295

OR: Odds’ ratio; GCS: Glasgow Coma Scale.

## Data Availability

The data presented in this study are available on request from the corresponding author. The data are not publicly available due to the restriction of local law and government policy.
